# Matrix Metalloproteinase 1 Is Necessary for the Migration of Human Bone Marrow-Derived Mesenchymal Stem Cells Toward Human Glioma

**DOI:** 10.1002/stem.50

**Published:** 2009-06

**Authors:** Ivy A W Ho, Kelly Y W Chan, Wai-Hoe Ng, Chang M Guo, Kam M Hui, Philip Cheang, Paula Y P Lam

**Affiliations:** a Laboratory of Cancer Gene Therapy, National Cancer CenterSingapore; b Division of BioEngineering, School of Chemical and Biomedical Engineering, Nanyang Technological UniversitySingapore; c Department of Neurosurgery, National Neuroscience InstituteSingapore; d Department of Orthopedic, Singapore General HospitalSingapore; e Bek Chai Heah Laboratory of Cancer Genomics, Division of Cellular and Molecular Research, National Cancer CenterSingapore

**Keywords:** Human mesenchymal stem cells, MMP1, Migration, HSV-1, Glioma

## Abstract

Human mesenchymal stem cells (MSCs) have increasingly been used as cellular vectors for the delivery of therapeutic genes to tumors. However, the precise mechanism of mobilization remains poorly defined. In this study, MSCs that expressed similar cell surface markers and exhibited multilineage differentiation potentials were isolated from various donors. Interestingly, different MSC isolates displayed differential migration ability toward human glioma cells. We hypothesized that distinct molecular signals may be involved in the varied tumor tropisms exhibited by different MSC isolates. To test this hypothesis, gene expression profiles of tumor-trophic MSCs were compared with those of non–tumor-trophic MSCs. Among the various differentially regulated genes, matrix metalloproteinase one (MMP1) gene expression and its protein activities were enhanced by 27-fold and 21-fold, respectively, in highly migrating MSCs compared with poorly migrating MSCs. By contrast, there was no change in the transcriptional levels of other MMPs. Functional inactivation of MMP1 abrogated the migratory potential of MSCs toward glioma-conditioned medium. Conversely, the nonmigratory phenotype of poorly migrating MSC could be rescued in the presence of either recombinant MMP1 or conditioned medium from the highly migrating MSCs. Ectopic expression of MMP1 in these poorly migrating cells also rendered the cells responsive to the signaling cues from the glioma cells in vivo. However, blocking the interaction of MMP1 and its cognate receptor PAR1 effectively diminished the migratory ability of MSCs. Taken together, this study provides, for the first time, supporting evidence that MMP1 is critically involved in the migration capacity of MSCs, acting through the MMP1/PAR1 axis. Stem Cells 2009;27:1366–1375

## INTRODUCTION

Human mesenchymal stem cells (MSCs) are nonhematopoietic adult stem cells with multipotent capacities. The innate tropism of MSCs for tumors, combined with the fact that these cells can be expanded to a clinical scale production with ease, have prompted great interest in using MSCs to deliver antitumor agents to the tumor microenvironment. This is of particular importance to targeting tumor cells with invasive capacity such as glioma cells. However, the mechanism and factors responsible for the tumor tropism of MSCs remain fully elucidated.

MSC migration has been postulated to be similar to hematopoietic stem cell (HSC) migration as both cell types reside in the bone marrow. One of the most widely recognized receptor/ligand pairs involved in HSC trafficking is the stromal cell derived factor-1 (SDF-1; also know as chemokine [C-X-C motif] ligand 12 or CXCL12) and its receptor, chemokine receptor four (CXCR4), which are crucial for the homing and engraftment activities of HSC [1–3] as well as for recruitment of endothelial progenitor cells to sites of ischemic tissues [4]. However, the role of SDF-1/CXCR4 in MSC mobilization is less clear compare with HSCs. In fact, recent studies have suggested that SDF-1/CXCR4 axis may not play a major role in MSC migration. Findings from Ip et al. demonstrated that the blocking of CXCR4 receptors had no impact on murine MSC migration [5]. Furthermore, CXCR4/SDF-1 does not possess the same migration importance in the ability of HSC to migrate and engraft in immunodeficient animals unless enforced expression of CXCR4 is employed [6,7]. This finding is recently confirmed in the context of MSCs in which enforced expression of CXCR4 by lentiviral gene transfer was demonstrated to enhance homing of MSCs to bone marrow in vivo [8]. Clearly, a better understanding of the mechanisms that regulate the migration abilities of MSCs is needed before these cells could be employed as potent agents for delivering therapeutic genes to tumor cells.

The signaling cues to mobilize MSCs appear to be dependent on the physiologic/pathologic status of the local environment. For example, the hepatocyte growth factor/c-met signaling pathway has been implicated in MSC mobilization and recruitment to damaged tissues [9,10]. Under hypoxic stress, MSCs exhibited an increase in the migratory propensity induced by basic fibroblast growth factor [11] and vascular endothelial growth factor in the damaged regions [12]. In the tumor microenvironment, recruitment of MSCs to tumor cells was associated with elevated expression of proinflammatory molecules such as interleukin-(IL) 6, IL-8, and monocyte chemoattractant protein-1, which may be mediated by the activation of urokinase plasminogen activator and its receptors on human tumor cells [13]. At present, it is unclear whether cytokines, chemokines, growth factors, or proteolytic enzymes activate the migratory process of MSCs. This is because many of the studies on MSC migration involved profiling the conditioned medium of MSCs that led to the identification of many candidate factors or targeted knockdown of genes which have been shown to play a role in cellular migration. We have taken a different approach by performing gene expression profiles on batched MSCs that exhibited high migratory activities versus those that migrated poorly. This strategy has allowed us to focus on genes whose products exert a significant influence on the migration of MSCs toward tumor cells. The candidate gene, matrix metalloproteinase one or MMP1, was found to be highly upregulated in highly migratory MSCs that exhibited the tumor trophic property and was chosen for subsequent studies.

Cellular migration is a complex process that involves the breakdown of extracellular matrix (ECM) detachment of cells from the basal membrane, migration of cells from original location, survival of cells during the migration process, intravasation into target tissue, and finally, interaction of the migrated cells with the target microenvironment [14]. The degradation of ECM during the migration process requires the action of proteolytic enzymes such as metalloproteinases (MMPs), which are zinc-dependent endopeptidases [15]. MMPs are secreted as inactive proenzymes or zymogens that are activated by the cleavage of the prodomain [16]. Depending on the substrate specificity and structure, they are divided into several subgroups: collagenases (e.g., MMP1), stromelysins (e.g., MMP3; MMP10), matrilysins (e.g., MMP7; MMP26), gelatinases (e.g., MMP2; MMP9), and membrane-type matrix metalloproteinase-1 (MT1-MMP). In particular, interstitial collagenase (MMP1) has been reported to be involved in the invasion of breast carcinoma [17]. Stromal-derived MMP1 was recently shown to cleave and activate the G-protein-coupled receptor, protease-activated receptor one (PAR1), leading to activation of intracellular signal that regulates the invasion process in breast cancer cells [18].

In the present study, the functional role of MMP1 in the migratory activities of various MSC isolates was studied. Targeted knockdown of endogenous MMP1 was shown to inhibit the migration ability of MSCs in vitro. Conversely, exogenous expression of MMP1 in poorly migrating MSCs could reconstitute the tumor trophic abilities of these cells in vitro and in vivo. In addition, the disruption of interaction between MMP1 and PAR1 was found to severely impair the migration ability of MSCs. Taken together, our results showed, for the first time, the functional importance of the MMP1/PAR1 axis in modulating the migration of MSCs toward human glioma.

## MATERIALS AND METHODS

### Cell Culture and RNAi Transfection

The Institutional Review Board of National Cancer Center and Singapore General Hospital have approved this study. Isolation and characterization of MSCs was performed as previously described [19]. Primary glioma was obtained from a brain tumor biopsy of a patient diagnosed with grade IV gliomas. Normal human astrocytes (NHA), ΔGli36, and 2-2 cells were cultured as described previously [19]. Full methods are available as supporting information.

RNAi transfection was performed using Lipofectamine RNAiMax (Invitrogen, Carlsbad, CA, http://www.invitrogen.com). Stealth negative control (medium GC; Invitrogen) was used as control. In brief, all RNAis were transfected at a final concentration of 20 nM into 1 × 10^5^ cells cultured in a six-well dish (BD Biosciences, Franklin Lakes, NJ, http://www.bdbiosciences.com) according to the manufacturer's protocol.

### Construction and Packaging of pHGCX-MMP1 Herpes Simplex Virus-1 Amplicon Viral Vector

The pHGCX-MMP1 Herpes Simplex Virus (HSV)-1 amplicon plasmid vector which contains the eGFP gene under the control of the viral immediate early promoter (IE4/5) was obtained from Dr. Y Saeki (Massachusetts General Hospital, Boston, MA). The MMP1 gene was inserted into the *Kpn*I and *Not*I site located downstream of the strong CMV promoter. See supporting information Methods for detail information.

### In Vitro Migration Assay

A Modified Boyden chamber assay was used to investigate the in vitro migration of MSCs. MSCs (1 × 10^4^) were cultured in a 24-well tissue culture insert with an 8 μm pore size membrane (BD Biosciences). Migration of MSCs across the membrane was subsequently determined by counting the number of propidium iodide-stained nuclei on the underside of the membrane under ×200 magnification. Full method is included in the supporting information Methods.

### In Vivo Migration Assay

MSCs (5 × 10^4^), suspended in 5 μl of complete medium, were injected into the contralateral hemisphere of ΔGli36 human glioma cells-bearing (2 × 10^5^) mice 7 days post-tumor implantation (Bregma [0, 0], 2.0 mm lateral, 2.5 mm depth). Migration of MSCs from the site of injection was assessed 2 weeks post-MSC cells implantation. Quantification of migrated MSC was performed on single-cell suspension using flow cytometry. See supporting information Methods for detail. All animal experiments were performed according to the guidelines and protocols approved by the Institutional Animal Care and Use Committee at the Singapore General Hospital.

### Varani Migration Assay

Cell migration was quantified by recording the number of cells migrated away from the agarose drops using a method described by Varani et al. [20]. MSCs were resuspended (1 × 10^7^ cells per milliliter) in culture medium containing 0.3% low melting point agarose and maintained at 37°C. Drops of cell suspensions of approximately 1.5–2 μl were applied to the center of the wells in a 24-well tissue culture dish. The dish was then placed on ice for 15 minutes to allow the agarose to solidify. The cell-laden agarose droplets were then slowly covered with 500 μl of either glioma-conditioned medium or fresh culture medium. Cell migration was measured daily for 2 days. Each sample was repeated in triplicate and the experiment was repeated twice. Migrated cells were visualized using wide-field microscopy with an inverted microscope (TE300; Nikon, Tokyo, Japan, http://www.nikon.com), and images were acquired on a CCD color digital camera (DXM1200F) using image acquisition software (ACT-1 v2.7; Nikon).

### Affymetrix GeneChip Analysis

The Affymetrix cDNA GeneChip Human Genome U133 Plus 2.0 Array which composed of 47,000 transcripts was used to identify factors that influence the migration of MSC. We compared the gene expression profiles of highly migratory MSCs (MSC-1; MSC-9) and lowly migratory MSCs (MSC-2; MSC-8). A total of two independent hybridizations were performed using cells of either passage 4 or 5. Five microgram of total RNA was converted into double-stranded cDNA using a T7-(dT)24 primer containing T7 RNA polymerase promoter and Superscript II reverse transcriptase (Affymetrix Inc., Santa Clara, CA, http://www.affymetrix.com). cRNA labeling, hybridizations, washes, and scan steps were performed according to manufacturer's instructions (Affymetrix Inc.). Probe arrays were scanned using the Affymetrix Microarray Suite, and images were imported as CEL files into Partek Genomic Suite (Partek Inc., St. Louis, MO, http://www.partek.com) for analysis. Genes of interest were matched to those in the Affymetrix NetAffix Gene Ontology analysis system. The expression microarray has been submitted to the Gene Expression Omnibus database at http://www.ncbi.nlm.nih.gov/geo/. The accession number is GSE12098.

### Real-Time Reverse Transcriptase Polymerase Chain Reaction, MMP1 ELISA, and Bioactivity Assay

Real-time reverse transcriptase polymerase chain reaction (RT-PCR) was performed as described previously [21]. Quantitation of pro-MMP1 protein expression and activity were performed using Quantikine human pro-MMP1 ELISA kit (R&D Systems Inc., Minneapolis, http://www.rndsystems.com) and MMP1 Biotrak activity assay system (GE Healthcare UK Limited, Little Chalfont, Buckinghamshire, U.K., http://www.gehealthcare.com) respectively, according to manufacturer's suggestions. See supporting information Methods for details.

### Statistical Analysis

Statistical analyses were performed using Prism 3.0 (Graphpad Software Inc., San Diego, CA, http://www.graphpad.com). Nonpaired parametric data were compared with *Student*'*s t*-test; for in vivo quantitation of migrated MSCs, paired *t*-test was used. *p* values <.05 were considered statistically significant.

## RESULTS

### Isolation and Tumor-Trophic Characterization of MSCs In Vitro and In Vivo

**Figure 1:**
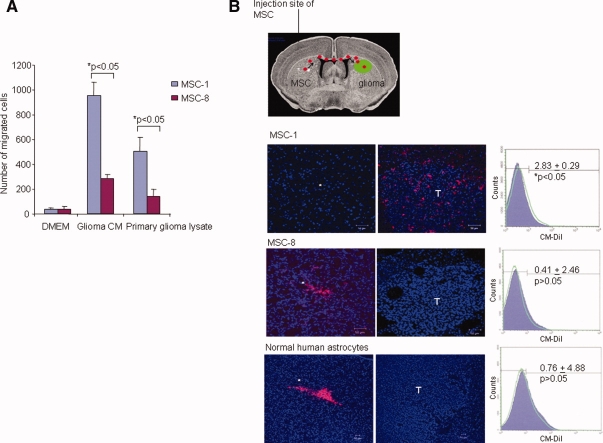
Differential migration properties of MSCs. **(A):** Migration of MSCs toward conditioned medium from glioma cells and primary glioma lysate was analyzed using a modified Boyden chamber assay. Migration of MSCs was determined by counting the number of propidium iodide-stained nuclei on the underside of the membrane under ×200 magnifications. Bar graph represents number of migrated cells. **(B):** Coronal section of the mouse brain indicating the injection site (*) of MSC, and the location of the preimplanted ΔGli36 human glioma cells (T). Confocal fluorescent images showed the migration of the MSC-1 (upper panel), MSC-8 (middle panel), and normal human astrocytes (lower panel) to the tumor sites. Images were taken 14 days post-CM-DiI-labeled-MSC injection with a confocal system (LSM 510 Meta; Carl Zeiss, Göttingen, Germany) using a ×20/0.75 N.A. Plan-Fluor objective. Right panel: flow cytometry analysis of the percentage of CM-DiI cells in the left and right hemisphere. Purple-filled curve indicates the percentage of cells in the left hemisphere; green line indicates the percentage of cells in the right hemisphere. Total number of cells used for fluorescence-activated cell sorting analysis was 1,000,000. Data shown are averages ± SEM, *n* = 4. Abbreviations: DMEM, Dulbecco's modified Eagle's medium; FITC, fluorescein isothiocyanate; MSCs, mesenchymal stem cells.

Human bone marrow-derived MSCs were isolated as previously described [19]. Both MSC-1 and MSC-8 were positive for CD13, CD44, CD90, and CD105, but were negative for the hematopoietic lineage markers CD34 (hematopoietic stem/progenitor cells), CD45 (leukocyte), and CD49a (monocytes and activated T-cells) (supporting information Fig. S1A). In addition, the abilities of MSCs to differentiate into adipocytes and osteocytes were demonstrated by the presence of Oil Red-O staining and von Kossa staining, respectively (supporting information Fig. S1B). Primary human glioma biopsy, in comparison with glioma cells lines, contains a variety of cells and tissue structures such as ECM, stroma, macrophages, and tumor cells, which closely resemble the in situ tumor scenario. Using a modified Boyden chamber assay, MSC-1 exhibited significant migration in the presence of either glioma-CM or primary glioma lysates (Fig. 1A). By contrast, the migratory activities mediated by MSC-8 were greatly impeded compared with MSC-1, suggesting that the differential migratory potential is an intrinsic property of MSCs. These results indicate that MSCs are chemoattracted toward soluble factors secreted by primary human brain tumor and glioma cell line. Furthermore, different MSC isolates exhibited differential degrees of tumor tropism in vitro.

To establish if similar behavior was observed in the in vivo setting, both MSC-1 and MSC-8 were prelabeled with a red fluorescent vital dye, CM-DiI, followed by implantation into the contralateral hemisphere of glioma-bearing mice (Fig. 1B). Two weeks after injection, with MSCs, these cells had migrated away from its original injection site and were found in the contralateral hemisphere, specifically around the peripheral and within the ΔGli36 tumor region. CM-DiI-labeled-MSC-1 could not be detected in the original injection site (i.e., the hemisphere opposite to the implanted ΔGli36 cells), thus confirming the tumor trophic property of MSC-1. Furthermore, the percentage of CM-DiI-positive MSC-1 in the glioma-bearing hemisphere was 2.83% ± 0.29% higher than the nonglioma-bearing hemisphere (Fig. 1B). On the contrary, similarly injected CM-DiI-labeled-MSC-8 and CM-DiI-labeled NHA were not detected at the glioma-bearing hemisphere as shown by both the immunofluorescence image and flow cytometry analysis. Both MSC-8 and NHA-injected hemisphere exhibited more CM-DiI positive cells in comparison with the glioma-bearing region. In fact, the cells remained near or at the injection site. These results suggested that different MSC isolates exhibited differential migration ability toward glioma cells.

### Differential Migratory Abilities of MSCs Is Mediated by MMP1

**Figure 2:**
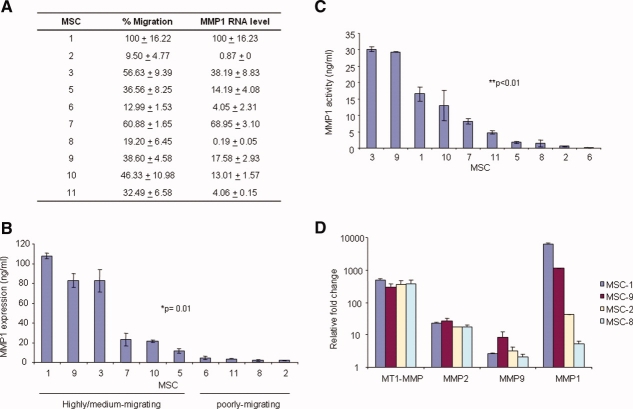
MMP1 is differentially expressed in various MSC isolates. **(A):** Differential migration of MSCs and real-time RT-PCR analysis of MMP1 transcript in MSCs. Data shown are averages of four replicates of independent experiments. Data shown for real-time RT-PCR are averages of duplicate samples, experiments were performed independently thrice. **(B):** Expression of MMP1 in 100 μg of conditioned medium harvested from various MSCs. Data shown are averages of duplicate wells from representative experiments performed independently thrice. **(C):** MMP1 activity was quantitated in conditioned medium harvested from various MSCs. Data shown are averages of duplicate wells ± SEM from representative experiments performed independently twice. **(D):** Real-time RT-PCR analyses of MMP1, MMP2, MMP9, and MT1-MMP transcripts were performed in highly migratory MSC-1 and MSC-9 versus poorly migrating MSC-2 and MSC-8. For comparison purposes, the fold change of each member of the MMP was expressed to the human embryonic kidney cells, 293. Data shown are averages of duplicate samples and performed independently twice. Abbreviations: MMP, matrix metalloproteinase; MSCs, mesenchymal stem cells; MT1, membrane type 1.

To examine if the observed differential migration ability was donor-specific, MSCs harvested from additional eight donors were tested for their migration activities toward glioma. These cells were morphologically and immunophenotypically similar to both MSC-1 and MSC-8. Migration assays were performed four times using cells restricted to either passage 4 or 5 in an attempt to minimize variation due to prolonged in vitro culture. As shown in Figure 2A, differential migration abilities were observed in the various MSC isolates. Based on the percentage of migrated cells, we classified the MSCs into three categories, namely, highly migrating (MSC-1 > 66.6%), medium migrating (33.3% ≤ MSC-3, 5, 7, 9, 10 ≤ 66.6%), and poorly migrating (MSC-2, 6, 8, 11 < 33.3%).

To identify the molecular pathways involved in the differential migration activity of MSCs, gene expression profile between the highly migratory and poorly migratory MSCs were determined by cDNA microarray using Affymetrix GeneChip Human U133 Plus 2.0 Array. MSCs from the highly migrating and medium migrating group (MSC-1 and 9) were compared with those from the poorly migrating group (MSC-2 and 8). Using Partek software analysis, 239 genes were found to be upregulated by at least twofold in the highly migrating/medium migrating MSCs. Many of these genes that matched to those in the Affymetrix NetAffix Gene Ontology analysis system belong to chemokines, metalloproteinases, and cell adhesion molecules, as represented in supporting information Table S1. Among these candidate genes, MMP1 was chosen for further study because it exhibited the greatest fold change (27-fold) with a significant *p* value of .0037. To determine if the migration activity of MSCs could be correlated to the expression of MMP1, quantitative real-time RT-PCR analysis was performed (Fig. 2A). The mean expression values of MMP1 when normalized to 18S were found to be high (between values of 13 and 100) in highly migrating or medium migrating groups. On the other hand, MMP1 transcripts were minimally detectable (between values of 0.19 and 4.1) in poorly migrating MSCs. To further confirm the differential expression of MMP1 in various MSC isolates, the level of MMP1 protein expression was quantified using an ELISA assay. As shown in Figure 2B and 2C, MMP1 expression (*p* = .01) and activity was significantly higher (*p* = .004) in the highly migrating MSCs as compared with the poorly migrating MSCs. Taken together, these results show that the differential migratory abilities of MSCs are correlated, in part, to the functional expression of MMP1.

MMP2, MMP9, and MT1-MMP have been shown to be essential for the invasive capacity of human MSCs [22]. As such, the mRNA expression of these genes was analyzed in representative cells from each group using real-time RT-PCR analysis. The levels of MMP2, MMP9, and MT1-MMP transcripts were found to be similar between the highly migrating (as represented by MSC-1 and MSC-9) and the poorly migrating cells (as represented by MSC-2 and MSC-8) (Fig. 2D). This finding was consistent with our microarray data in that MMP2, MMP9, and MT1-MMP1 were not differentially expressed between the two groups of cells. Taken together, these results indicate that MMP2, MMP9, and MT1-MMP1 are not critical determinants of MSC migration.

### Targeted Knockdown of MMP1 RNA Inhibits Migration and Activity in MSCs

**Figure 3:**
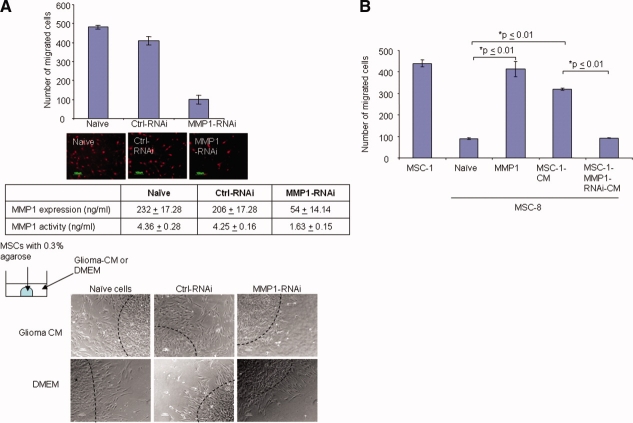
Targeted knockdown of MMP1 RNA inhibits migration and activity in MSCs. **(A):** Migration of MMP1-RNAi-transfected MSC was analyzed using a modified Boyden chamber assay. Bar graph represents number of migrated cells. Fluorescent images below showed representative images of PI-stained cells. Phase contrast photomicrograph showed the migration of MMP1-RNAi-transfected MSCs performed using the Varani migration assay, as representatively shown as original magnification ×100. All images were visualized using wide-field microscopy with an inverted microscope (TE300; Nikon), and images were acquired on a CCD color digital camera (DXM1200F; Nikon) using image acquisition software, ACT-1 v2.7 (Nikon). **(B):** Effect of exogenous MMP1 on the migration profile of poorly migrating MSC-8. Bar graph represents number of migrated cells. In all of the above experiments, data shown are averages of triplicates ± SEM from representative experiment. Abbreviations: CM, conditioned medium; Ctrl, control; DMEM, Dulbecco's modified Eagle's medium; MMP1, matrix metalloproteinase one; MSCs, mesenchymal stem cells; RNAi, RNA interference.

To further confirm the critical role of MMP1 in MSC migration, RNA interference assays were performed. Three MMP1-RNAi, namely, MMP1-RNAi-1, MMP1-RNAi-2, and MMP1-RNAi-3, that target different regions on the MMP1 transcript were synthesized. As shown in supporting information Figure S2A, these RNAi showed at least 75% reduction in the number of migrated cells when compared with control-RNAi (ctrl-RNAi)-transfected cells. To ensure an effective MMP1 RNA inhibition, these RNAi were pooled together and chosen for subsequent studies (supporting information Fig. S2A). To confirm the target specificity of the MMP1-RNAi, the endogenous transcriptional levels of MMP1, MMP2, MMP9, and MT1-MMP in MSC-1 were determined by real-time PCR. As shown in supporting information Figure S2B, MMP1-RNAi-MSC-1 had at least a 90% knockdown of MMP1 mRNA expression, which was not observed in ctrl-RNAi-treated-MSC-1 cells. In addition, in MSC-1 cells treated with either MMP1-RNAi or ctrl-RNAi, there was no significant decrease in the transcriptional levels of MMP2, MMP9, and MT1-MMP, thus confirming the specificity of RNAi against MMP1. The targeted knockdown of MMP1 gene resulted in corresponding decrease in the level of MMP1 protein and activity (Fig. 3A). As a consequence, the number of migrated cells from MMP1-RNAi-MSC-1 was significantly reduced when compared with those that were transfected with ctrl-RNAi or naïve cells. Similar results were obtained in other highly/medium migrating MSCs, namely, MSC-3, 5, 7, and 9, which have been transfected with MMP1-RNAi (supporting information Fig. S2C).

The role of MMP1 in the migration of MSC-1 toward human glioma was further investigated using Varani migration assay. MMP1-RNAi-MSC-1 cells showed an inhibited outward dissemination in the presence of glioma-CM, which was not observed in ctrl-RNAi-MSC-1 or fresh tissue culture medium (Fig. 3A). These results provide supporting evidence that the migratory activities of MSCs are mediated, at least in part, by MMP1.

### Impaired Migration of MSCs Can Be Rescued by Exogenous MMP1 Expression

As MMP1 is a secreted protein, we next ask whether the conditioned medium derived from MSC-1 (MSC-1-CM) could provide the necessary components to induce migration of the poorly migrating MSCs (MSC-8 and MSC-2). Our results showed that MSC-1-CM could effectively rescue the poorly migratory phenotype of MSC-8 and MSC-2 (Fig. 3B and supporting information Fig. S3A, respectively), with an enhanced in migratory activities by at least fourfold. Similarly, addition of exogenous recombinant human MMP1 proteins to MSC-8 and MSC-2 could significantly induce the migration of these cells. By contrast, conditioned medium from MMP1-RNAi-MSC-1 failed to induce migration of MSC-8 and MSC-2.

**Figure 4:**
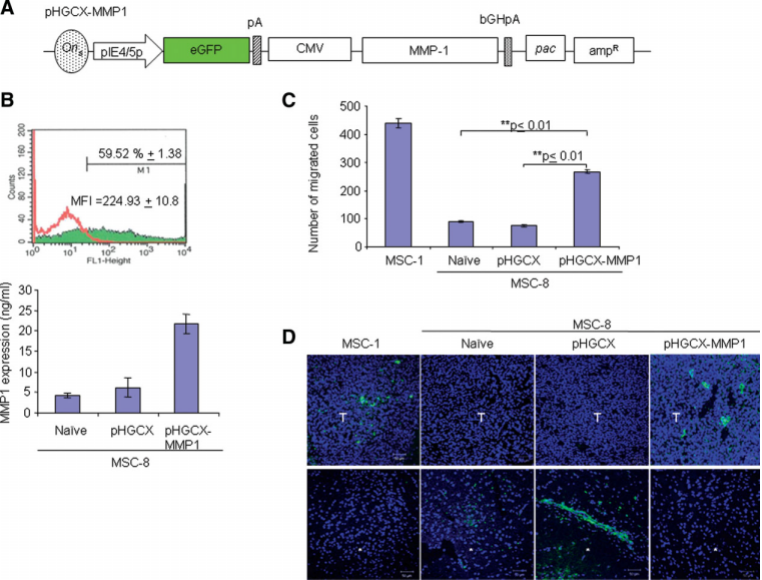
Overexpression of MMP1 restores the migration of MSC-8. **(A):** MMP1 gene was inserted into the multiple cloning site located downstream of the strong CMV promoter on the pHGCX-MMP1 HSV-1 amplicon vector, which contains the eGFP gene. **(B):** Top: Percentage of infectivity was determined using FACS for eGFP expression 18 hours postinfection. Flow cytometry FL-1 height analysis demonstrating the shift in peak of eGFP+ cells at MOI of 2.0. Red line represents mock transduced-MSCs; green-filled peak represents pHGCX-MMP1-transduced MSCs. Bottom: MMP1 expression in pHGCX-MMP1-transduced MSC was determined after 48 hours of infection at MOI of 2.0. Data shown are averages of triplicates ± SEM from representative experiment. **(C):** Migration of pHGCX-MMP1-transduced-MSC-8 was analyzed 48 hours postinfection using a modified Boyden chamber assay. Bar graph represents number of migrated cells. Data shown are averages of triplicates ± SEM, experiment was performed independently twice. **(D):** In vivo migration of pHGCX-MMP1-transduced-MSC-8 in glioma-bearing mice. Confocal fluorescent images showed the migration of the CM-DiI-labeled naïve MSC-1, CM-DiI-labeled naïve MSC-8, pHGCX-transduced-MSC-8, and pHGCX-MMP1-transduced-MSC-8 to the tumor sites. Both CM-DiI-labeled naïve MSC-1 and CM-DiI-labeled naïve MSC-8 were pseudocolored green. Images were taken 14 days post-CM-DiI-labeled-MSC injection with a confocal system (LSM 510 Meta; Carl Zeiss, Göttingen, Germany) using a ×20/0.75 Numerical Aperture (N.A.) Plan-Fluor objective. Sections were shown at original magnification ×200. *, Injection site, T, tumor. Abbreviations: amp, ampicillin; bGHpA, bovine growth hormone poly A; CMV, cytomegalovirus; eGFP, enhanced green fluorescent protein; MFI, mean fluorescence intensity; MMP1, matrix metalloproteinase one; MSCs, mesenchymal stem cells; pac, packaging signal.

In view of the above observations, a gain-of-function assay was performed to evaluate if overexpression of MMP1 would potentiate the migration of MSC-8 and MSC-2 toward glioma-CM. We have previously demonstrated that MSCs could be transduced efficiently by a HSV-1 amplicon viral vector while retaining its stem cell characteristics [19]. To further confirm the functional involvement of MMP1 in MSC migration, the gene encoding MMP1 was subcloned into a HSV-1 amplicon vector (denoted as pHGCX-MMP1) and subsequently packaged into recombinant virions (Fig. 4A). Poorly migrating MSC, MSC-8, and MSC-2 (shown in supporting information Figure) were used for the gain-of-function assay. Poorly migrating MSC-8 was efficiently infected by pHGCX-MMP1. As shown in Figure 4B, using multiplicity of infection (MOI) of 2.0, approximately 59% of enhanced green fluorescent protein (eGFP)+ cells could be detected after 18 hours of infection by FACS analysis. ELISA performed on the conditioned medium harvested at 48 hours postinfection confirmed the ectopic expression of MMP1 in pHGCX-MMP1-infected cells in comparison with controls (Fig. 4B). Transwell migration assay subsequently showed a significant increase in the percentage of pHGCX-MMP1-infected-MSC-8 and -2 that had migrated across the filter membrane, *p* < .01 (Fig. 4C and supporting information Fig. S3B). By contrast, MSC transduced with vector alone did not exhibit significant migration compare with naïve MSC-8. Taken together, these results confirm the important role of MMP-1 in mediating the MSC migration.

The effect of MMP1 overexpression on MSC tumor-tracking ability was further confirmed in vivo. The highly migrating MSC-1 was used as positive control and the poorly migrating naïve MSC-8 was used as a negative control. Fourteen days after injecting MSCs into mice bearing intracranial tumors, the brains were harvested and cryosectioned at 10 μm thickness. Our results showed that the reconstitution of MMP1 in MSC-8 could rescue the nonmigratory phenotype of MSC-8 as indicated by the presence of eGFP positive MSCs in the tumor region (denoted as T in Fig. 4D). On the contrary, pHGCX-transduced-MSC-8 remained close to the injection site (indicated as “*” in Fig. 4D), exhibiting similar characteristics as the naïve MSC-8. These results indicated that MMP1 could sensitize poorly migrating MSCs to signaling cue from glioma cells. Taken together, we have clearly demonstrated that MSCs with minimal migratory activities can be reverted by supplementing the cells with recombinant or exogenous MMP1 gene products, which are essential for the tumor-trophic migratory activities of MSCs.

### Interference of PAR1/MMP1 Axis in Highly Migratory MSCs Completely Abolish the Migratory Activities

**Figure 5:**
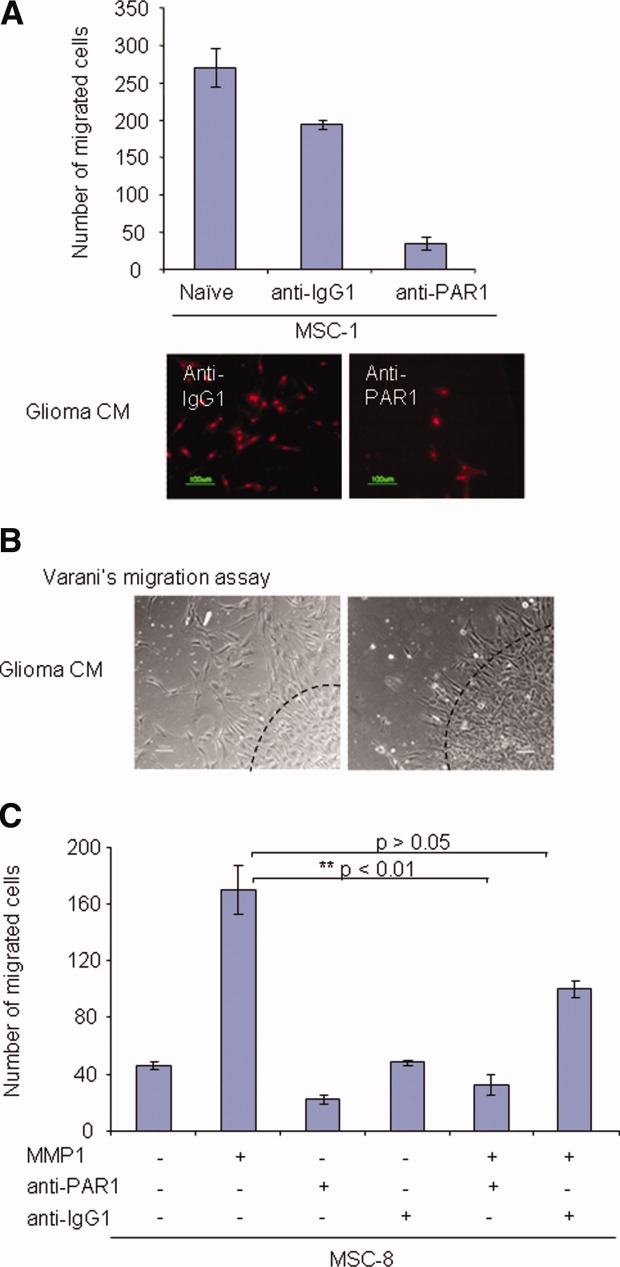
Functional MMP1/PAR1 axis mediates MSC migration. **(A):** Effect of anti-PAR1 blocking antibody on the migration of MSC-1 was examined using a modified Boyden chamber assay. Bar graph represents number of migrated cells. Data shown are averages of triplicates ± SEM, experiment was repeated independently three times. Photomicrograph showed the representative images of PI+ migrated cells at original magnification ×200. **(B):** Varani migration assay was performed to confirm the effect of PAR1 on MSC migration. Photomicrograph showed migration of MMP1-RNAi-transfected MSCs. Representative images from two independent experiments were shown. Images were shown as original magnification x100. Slides were visualized using wide-field microscopy with an inverted microscope (TE300; Nikon), and images were acquired on a CCD color digital camera (DXM1200F; Nikon) using image acquisition software, ACT-1 v2.7 (Nikon). **(C):** Effect of MMP1/PAR1 interaction on the migration ability of MSC-8 treated with the various proteins was examined using a modified Boyden chamber assay. Bar graph represents number of migrated cells. Data shown are averages of triplicates ± SEM, experiment was performed independently twice. Abbreviations: CM, conditioned medium; MSCs, mesenchymal stem cells; PAR1, protease-activated receptor one.

Recently, the G protein-coupled receptor, PAR1, has been found to be cleaved by MMP1, which promotes breast cancer migration and invasion [18]. To investigate whether PAR1 plays a role in MMP1-dependent chemotaxis of MSCs, we examined whether the inhibition of PAR1 proteolysis may affect the ability of MSC-1 to migrate toward glioma-CM. The inhibition of PAR1 activation was performed by incubating MSC-1 in the presence of an anti-PAR1 monoclonal antibody (ATAP2), which specifically binds to the cleavage domain of PAR1, thus preventing the proteolysis of PAR1 by MMP1. Anti-PAR1 treated MSC-1 resulted in an approximately 85% decrease in the number of MSC-1 migrating to the glioma-CM in comparison with naïve MSC-1 (Fig. 5A). Conversely, no significant difference was observed in control IgG1-treated cells. This observation was further confirmed by the Varani migration assay in which anti-PAR1-treated MSC-1 failed to migrate toward glioma-CM (Fig. 5B). Similar experiments were also performed in poorly migrating MSC-8. The addition of anti-PAR1 antibodies, but not anti-IgG1, was shown to further reduce the mobility of MSC-8 toward glioma-CM. The addition of exogenous MMP1 to this anti-PAR1 treated MSC-8 also did not enhance the migratory activities (Fig. 5C). This suppressive effect on the migration of MSC-8 was lifted when exogenous MMP1 was added to the cells without the presence of PAR1 antibodies, thus demonstrating that MMP1-mediated MSC migration is directed solely through interaction with its cognate receptor, PAR1.

## DISCUSSION

It is becoming increasing clear that achieving targeted trafficking of stem cells will be critical for effective tissue regeneration in the clinic. In this study, we have demonstrated that MSCs exhibit differential tumor tropisms despite the fact that they were isolated using an identical procedure and cannot be distinguished based on their phenotypic or multipotential characteristics. The isolation of MSCs with different tumor-trophic properties has allowed us to identify the molecular characteristics of migrating cells in tumor microenvironment. In particular, the differential migratory activities of MSCs are mediated, at least partially, by endogenous MMP1 expression.

Cell migration assays were performed to determine the gliomatrophic properties of our various MSC isolates. Interestingly, we observed that different MSC isolates exhibited differential migratory activities in vitro, which could be grouped according to their migratory activities into high, medium, and poor. This is unlikely due to higher cell culture confluence as reported by De Becker et al. [23], as both highly migrating and poorly migrating MSCs were seeded under a mean cellular density that was considered as high according to the reported experimental conditions. Furthermore, the differential tumor tropisms exhibited by MSCs was clearly demonstrated in intracranial human glioma xenografts (Fig. 1B). At present, we are uncertain if the differential migration activities exhibited by the various isolates of MSCs reflected the *bona fide* pathophysiological state in which the MSCs are isolated or merely that the pool of MSCs contain subpopulations at different states of differentiation that are indistinguishable by cell surface markers. Nevertheless, the identification of these cells has provided us with an approach to identify distinct signals that are critical to the migration of MSCs by comparing the two MSC populations in comparative gene expression studies. By contrast, many of the previous reports have focused on identifying genes involved with MSC migratory activities by exposing MSCs to different stimuli or tumor CM before gene profile expression assays [24,25].

Using DNA microarray analysis, our results showed that MMP1 is significantly upregulated (∼27-fold) in highly migrating MSCs in comparison with the poorly migrating MSCs. This was confirmed by the ∼10-fold to 1,000-fold higher level of MMP1 transcripts in the highly migratory MSCs compare with poorly migratory MSCs using real-time PCR. The differential mRNA expression of MMP1 was further supported by ELISA assay: MSCs with greater migratory activities were shown to secrete substantial amounts of MMP1 proteins into the culture supernatants (Fig. 2B). In addition, the level of active MMP1 was found to be tightly correlated with the migratory activities of various MSC isolates (Fig. 2C). Ideally, the relative amount of MMP1 RNA transcripts, MMP1 protein expression and functional cell migratory activities should be consistent among the various MSC isolates. For example, the top three MSC isolates with the highest migratory activities were MSC-1 (100 ± 16.22), MSC-7 (60.88 ± 1.65), and MSC-3 (56.63 ± 9.39). The MMP1 RNA levels were also found to be in the same order (Fig. 2A). However, the level of MMP1 protein expression was found to be highest from MSC-1, followed by that found in MSC-9 and MSC-3 (Fig. 2B). These interexperimental variations could be explained as each of the assays (RNA, protein, or cell migration) was performed at different times even though special care was taken to ensure that all experiments were performed using cells of passage 4 to 5. Nevertheless, the overall MMP1 mRNA, protein, and migration profiles were consistent among the highly migrating versus the poorly migrating MSCs.

MMPs are generally known for their roles in tissue remodeling by degradation of ECM and basement membrane components. However, MMPs have also been implicated in the activation of growth factors and cytokines by degrading their precursors or inhibitors, thereby adjusting the cancer cells to the tissue microenvironment [26,27]. MMP1 has been shown to degrade insulin-like growth factor binding proteins (IGFBP)-3 and -5 [28,29]. This in turn modulates the bioavailability of the insulin-like growth factors (IGFs) depending on tissue types and physiologic/pathologic status [30]. For example, MMP9 induced IGFBP2-IGF2 complex proteolysis resulted in the extracellular release of free IGF2 with positive and biologic effect on astrocytoma cellular growth and migration [30]. From our microarray results, the level of IGF2 transcripts was the second highest in MSCs with greater mobility in comparison with those that migrate poorly (supporting information Table S1). Thus, it is possible that MMP1 could also act as an IGFBP2 proteinase; the observed elevated level of IGF2 could represent the released, unbound IGF2 that ultimately initiates the mobilization of MSCs. Alternatively, the migration process may also be guided by cytokines, such as IL-8, and chemokines, such as CXCL1 and CXCL2, all of which have been found to be elevated in the highly migratory MSC population. The precise signal transduction pathway that governs the migration of MSCs will require further study.

Interestingly, even though platelet derived growth factor β (also known as PDGF-BB), MMP2, MMP9, and MT1-MMP were reported to be players mediating the migration and invasion of MSCs in vitro [22,31], upregulation of these genes in highly migratory MSCs was not observed. Similarly, there was no significant difference in the level of MMP2, MMP9, and MT1-MMP transcripts expression between the highly migratory (MSC-1 and MSC-9) versus poorly migratory MSCs (MSC-2 and MSC-8) when analyzed using real-time RT-PCR (Fig. 2). However, the design of this experimental study does not take into account the possible synergistic effect of these MMPs in the regulation of MSC mobilization. Hence, we could not exclude that these MMPs may still play a role in the overall mobilization of MSCs.

Recently, Boire et al. reported that PAR1 is a MMP1 receptor that promote invasion and tumorigenesis of breast cancer cells in vitro and in vivo [18]. Shi et al. showed that blocking PAR1 cleavage and activation using anti-PAR1 antibody could inhibit invasion and chemotaxis of prostate cancer cells [32]. Using a monoclonal antibody against PAR1, we showed that blocking the MMP1/PAR1 interaction significantly reduced the migration ability of MSCs. The importance of this axis in MSC migration was further supported by the lack of migratory response when recombinant MMP1 was added to cells previously incubated in anti-PAR1 antibody. Furthermore, Western blot analysis showed that the PAR1 expression level was similar in highly migrating versus poorly migrating MSCs (supporting information Fig. S4). Thus, it appears that the level of MMP1 expression and the specific interaction of MMP1 with PAR1 proteins determine the differential migration ability of MSCs. In bone marrow-derived MSCs, the level of MMP1 was reported to be regulated by the hypoxia-inducible factor-1α [33]. Ectopic expression of the secretory form of fibroblast growth factor-1 could also induce MMP1 transcription in endothelial cells, thus resulting in enhanced migratory activities [34]. On the other hand, PAR1 activation in human late endothelial progenitor cells has been shown to enhance the mRNA levels of both SDF-1 and CXCR4 [35]. We have not studied the SDF-1/CXCR4 axis closely because these genes were not differentially expressed between the highly migrating and poorly migrating MSCs. This finding might explain the observation why only a small subpopulation of human MSC has been shown to express functional CXCR4 receptors [36]. However, we could not exclude these genes from providing secondary support that may assist in the mobilization of MSCs. At present, the potential role of MMP1 in regulating SDF-1 expression is unknown; it is also unclear whether MMP1/PAR1 axis acts directly or indirectly on SDF-1 and/or CXCR4 activation. As such, further research is necessary to dissect the relation between the two pathways. Nevertheless, it is becoming clear that both the ECM and the microenvironment surrounding MSCs are not merely scaffold, but also harbor cryptic biological functions that may regulate the migration, plasticity, self-renewal and pluripotency of MSCs.

In conclusion, we report that the migratory activity of MSCs toward glioma is mediated via the MMP1/PAR1 axis in vitro and in vivo. An adequate understanding of the tumor tropism of MSC bears important implication for effective cellular delivery of therapeutic agents for brain tumor therapy.

## CONCLUSION

Our results highlighted the critical role that MMP1 plays in the process of mobilizing MSCs toward human glioma cells. MSCs expressing low levels of MMP1 fail to response to signaling cues from human glioma cells even though other MMPs are present at levels similar to the highly migrating MSCs. Targeted knockdown of MMP1 or the inactivation of PAR1 that disrupt its association with MMP1 resulted in the lost of MSC migratory activities. Anti-PAR1 antibodies treated MSCs could not migrate even in the presence of exogenous MMP1, suggesting that both MMP1 levels and the specific interaction between MMP1 and PAR1 are important factors that determine the migratory abilities of MSCs. To our knowledge, this is the first comprehensive report on the involvement of MMP1 on the migration of MSCs via the activation of the PAR1 receptor.

## DISCLOSURE OF POTENTIAL CONFLICTS OF INTEREST

The authors indicate no potential conflicts of interest.
