# Supplementary material for: Matrix Metalloproteinase 1 Is Necessary for the Migration of Human Bone Marrow-Derived Mesenchymal Stem Cells Toward Human Glioma
Source: Stem Cells. 2009 Jun;27(6):1366–75. doi: 10.1002/stem.50 (PMC2771102; doi:10.1002/stem.50)
Supplement: Supplementary file 1 [file stem0027-1366-SD1.doc]

**Table S1. Genes upregulated in highly-migrating MSC in comparison to poorly-migrating MSC.**

| **Probeset ID** | **Gene Title** | **Gene Symbol** | **Fold change** | **GO biological process** |
| --- | --- | --- | --- | --- |
| 204475_at | matrix metallopeptidase 1 (interstitial collagenase) | MMP1 | 27.2 | Metalloendopeptidase, proteolysis |
| 202409_at | insulin-like growth factor 2 (somatomedin A) | IGF2 | 6.0 | Cell proliferation, cell surface receptor-linked signalling, cell death |
| 204470_at | chemokine (C-X-C motif) ligand 1 | CXCL1 | 4.9 | Chemotaxis, cell proliferation, immune response |
| 209774_x_at | chemokine (C-X-C motif) ligand 2 | CXCL2 | 4.5 | Chemotaxis, immune response |
| 212865_s_at | collagen, type XIV, alpha 1 (undulin) | COL14A1 | 3.0 | Cell adhesion, ECM organization |
| 217901_at | Desmoglein 2 | DSG2 | 2.85 | Cell adhesion |
| 206025_s_at | tumor necrosis factor, alpha-induced protein 6 | TNFAIP6 | 2.7 | Cell adhesion, immune response |
| 216379_x_at | CD24 molecule | CD24 | 2.6 | Cell activation, regulation of cytokine and chemokine pathways, Wnt receptor signaling, cell migration |
| 1552627_a_at | Rho GTPase activating protein 5 | ARHGAP5 | 2.4 | Cell adhesion |
| 202859_x_at | interleukin 8 | IL8 | 2.3 | Angiogenesis, cell adhesion, chemotaxis, cell motility, immune response |
| 209031_at | Cell adhesion molecule 1 | CADM1 | 2.3 | Cell adhesion, cell recognition, immune response, apoptosis |
| 1555167_s_at | pre-B-cell colony enhancing factor 1 | PBEF1 | 2.3 | Cell proliferation, cytokine |
| 207370_at | integrin-binding sialoprotein (bone sialoprotein, bone sialoprotein II) | IBSP | 2.2 | Cell adhesion, ossification |
| 1565823_at | Septin 7 | SEPT7 | 2.2 | Cell cycle, cytokineses, protein hetero-oligomerization |
