# Supplementary material for: Matrix Metalloproteinase 1 Is Necessary for the Migration of Human Bone Marrow-Derived Mesenchymal Stem Cells Toward Human Glioma
Source: Stem Cells. 2009 Jun;27(6):1366–75. doi: 10.1002/stem.50 (PMC2771102; doi:10.1002/stem.50)
Supplement: Supplementary file 2 [file stem0027-1366-SD2.doc]

**Table S2 Primers used for characterization**

| **Primer** | **Sequence** | **Product size** | **Annealing temp (°C)** |
| --- | --- | --- | --- |
| MMP1 | 5’-ATGCTGAAACCCTGAAGGTG-3’  R 5’-CTGCTTGACCCTCAGAGACC-3’ | 234 | 55 |
| MMP9 | F-5’-CACTGTCCACCCCTCAGAGC-3’  R-5’-GCCACTTGTCGGCGATAAGG-3’ | 263 | 50 |
| MMP2 | F-5’-atgacagctgcaccactgag-3’  R-5’-atttgttgcccaggaaagtg-3’ | 174 | 50 |
| MT1-MMP | F 5’-cagagaaggcacacaaacga-3’  R-5’-cactggtgagacaggcttga-3 | 172 | 50 |
| 18S | F-5’-CCTGCGGCTTAATTTGACTC-3’  R-5’-CGCTGAGCCAGTCAGTGTAG-3’ | 320 | 55 |

RNAi sequence targeted against MMP1

RNAi-1-UCAACCACUGGGCCACUAUUUCUCC,

RNAi-2-AUCAAUGUCAUCCUGAGCUAGCUGA,

RNAi-3-AAUGAAAUUGAGCUCAACUUCCGGG.
