# Supplementary figures and images for: Matrix Metalloproteinase 1 Is Necessary for the Migration of Human Bone Marrow-Derived Mesenchymal Stem Cells Toward Human Glioma
Source: Stem Cells. 2009 Jun;27(6):1366–75. doi: 10.1002/stem.50 (PMC2771102; doi:10.1002/stem.50)

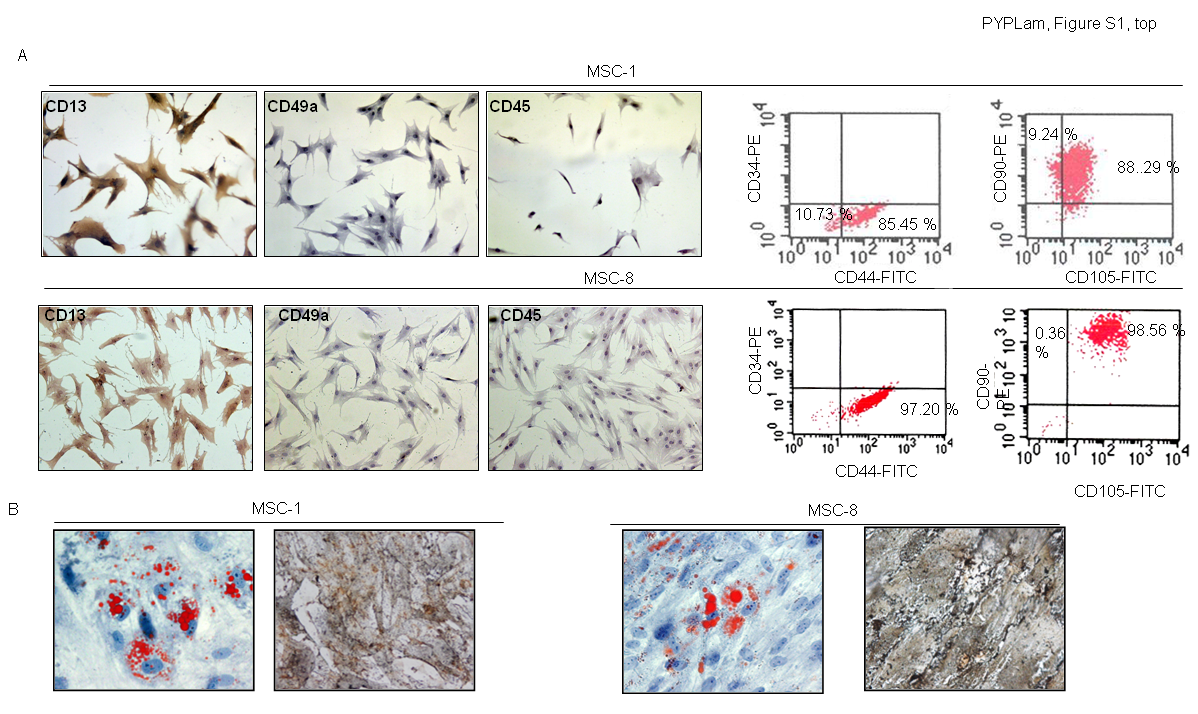

Supplement: Supplementary file 3 [file stem0027-1366-SD3.tif]

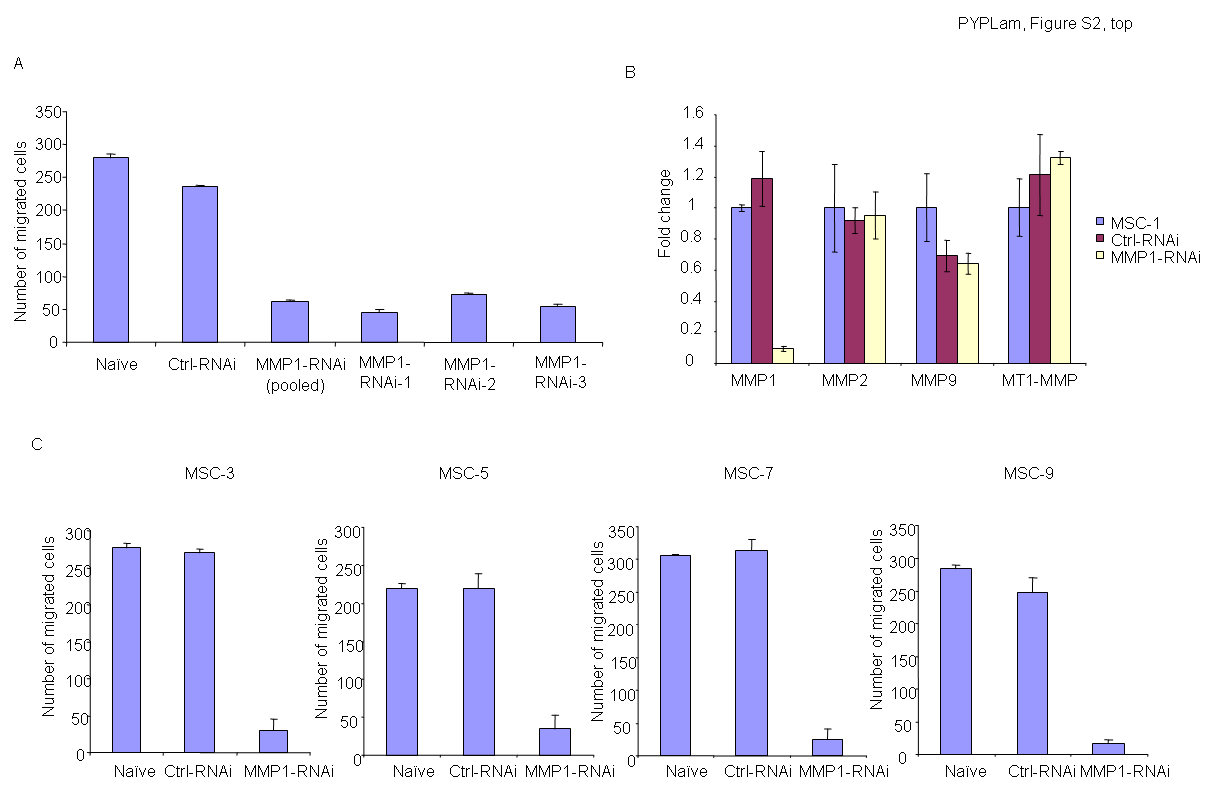

Supplement: Supplementary file 4 [file stem0027-1366-SD4.tif]

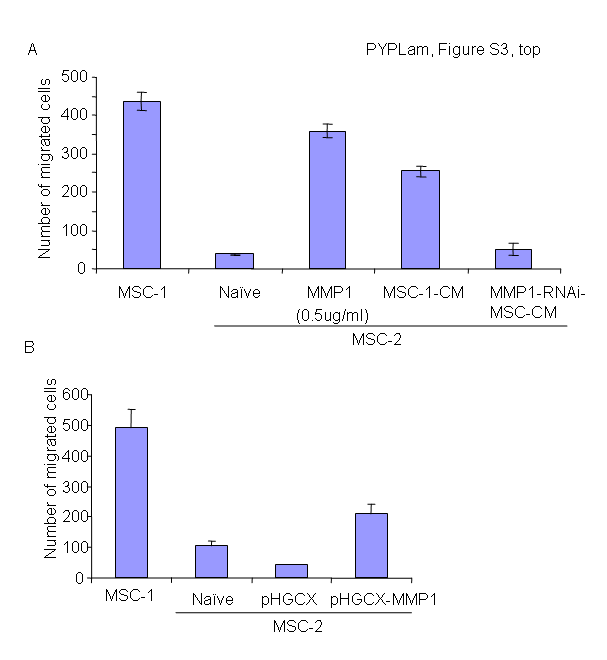

Supplement: Supplementary file 5 [file stem0027-1366-SD5.tif]

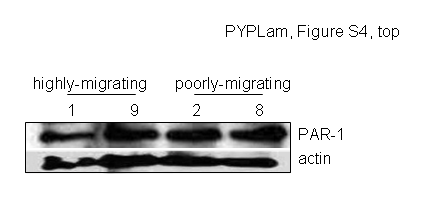

Supplement: Supplementary file 6 [file stem0027-1366-SD6.tif]
